# Elevated protein kinase C alpha expression may be predictive of tamoxifen treatment failure

**DOI:** 10.1038/sj.bjc.6600923

**Published:** 2003-04-29

**Authors:** D A Tonetti, M Morrow, N Kidwai, A Gupta, S Badve

**Affiliations:** 1 Department of Biopharmaceutical Sciences, University of Illinois at Chicago, Chicago, IL 60612, USA; 2 Department of Surgery, Northwestern University, Chicago, IL 60611, USA; 3 Department of Pathology, Northwestern University, Chicago, IL 60611, USA; 4 Department of Pathology, Indiana University, Indianapolis, IN 46202, USA

**Keywords:** breast cancer, tamoxifen resistance, protein kinase C alpha

## Abstract

We previously reported that stable transfection of protein kinase C alpha (PKC*α*) into T47D human breast cancer cells results in tamoxifen (TAM)-resistant tumour growth. Relevance of PKC*α* expression in clinical specimens was determined by comparing PKC*α* expression in tumours from patients exhibiting disease recurrence with patients remaining disease-free following TAM treatment. Our results suggest that PKC*α* expression may predict TAM treatment failure.

Resistance to tamoxifen (TAM), the endocrine treatment of choice for all stages of breast cancer, represents a significant clinical problem in the management of the disease. Identification of the key factors involved in the molecular mechanism of TAM resistance will undoubtedly lead to the development of logical therapeutic targets. It is well documented that the oestrogen receptor (ER) and protein kinase C (PKC) activity and abundance are inversely related in breast cancer cell lines and that PKC is elevated in malignant but not normal breast tissue (Borner *et al*, 1987; O'Brian *et al*, 1989; Gordge *et al*, 1996). Furthermore, increased activator protein-1 (AP-1) activity occurs in hormone-independent breast cancer cell lines and tumours (Dumont *et al*, 1996; Johnston *et al*, 1999; Schiff *et al*, 2000). Protein kinase C is an upstream activator of the AP-1 pathway. We reported that stable transfection of PKC*α* into T47D human breast cancer cells results in a hormone-independent phenotype and TAM-resistant tumour growth (Tonetti *et al*, 2000). Tumours formed from these cells grow in the presence of TAM and regress upon 17*β*-oestradiol (E2) administration (Chisamore *et al*, 2001). Our finding that both E2 and the pure antioestrogen, ICI 182,780, can inhibit these TAM-resistant tumours may now allow us to predict the efficacy of endocrine therapy. For example, tumours overexpressing PKC*α* may be stimulated to grow if the patient is treated with TAM, and therefore a more appropriate therapy may be an oestrogen-like compound or a pure antioestrogen. However, our T47D/PKC*α* tumour model cannot determine whether PKC*α* overexpression occurs in patients prior to TAM exposure, or is a result of long-term TAM treatment. To address this question, we identified paired paraffin-embedded tumour blocks from patients with primary and recurrent tumour samples that were available from the database of the Lynn Sage Breast Center of Northwestern Memorial Hospital. In addition, primary biopsies from patients remaining disease-free with at least 5 years of follow-up were identified. To determine changes, if any, in the intensity and/or incidence of PKC*α* expression, immunohistochemistry was performed on all biopsies.

## MATERIALS AND METHODS

### Tumour specimens

Paired paraffin-embedded tumour blocks from 15 patients where primary and recurrent tumour samples were identified from the database of the Lynn Sage Breast Center of Northwestern Memorial Hospital. In addition, 15 primary biopsies from patients remaining disease-free with at least 5 years of follow-up were identified. This study was approved by the Institutional Review Board at Northwestern University Medical School.

### Immunohistochemistry

Sections, 4 *μ*m thick, were deparaffinised with 100% CitriSolv dipped in 100% ethanol. Endogenous peroxidase activity was blocked with 0.3% H_2_O_2_ in methanol for 30 min, followed by immersion in graded alcohols. After rinsing in distilled water, antigen retrieval was accomplished by boiling in citrate buffer. After rinsing with PBS, the sections were blocked for 1 h with normal goat serum. The sections were incubated overnight at 4^o^C with a PKC*α* primary polyclonal antibody (C-20, Santa Cruz Biotechnology Inc., Santa Cruz, CA, USA). The sections were incubated for 30 min with biotinylated goat anti-rabbit IgG (H+L), followed by incubation for 30 min with HRP–Streptavidin (HistoMark Biotin Streptavidin kit; KPL, Gaithersburg, MD, USA). Sections were rinsed with PBS for 5 min between each reaction. 3,3′-Diaminobenzidine (DAB+) solution (DAKO, Carpinteria, CA, USA) was used as a chromogen. Finally, sections were counterstained with haematoxylin. Stained sections were photographed at × 40 magnification using an Olympus BX40 microscope attached to a SONY DP10 digital camera. Analysis of the entire slide was performed to score the intensity of staining. Intensity was evaluated semiquantitatively by assigning the score of either negative (0), weak positive (1+, <10% staining), moderate (2+, 10–30% staining), or intense (3+, >30% staining). The specificity of the staining was ascertained by several methods, including the use of PBS or isotypic nonspecific antibodies *in lieu* of the specific anti-PKC-*α* antibody. Dilution experiments using the primary antibody to assess specificity and observe extinction of signal were also performed. In addition, staining with the PKC*α* antibody was repeated in the presence of a specific PKC*α* blocking peptide (sc-3007, Santa Cruz, USA). There was no staining of the breast epithelial cells under these conditions.

## RESULTS

**Figure 1:**
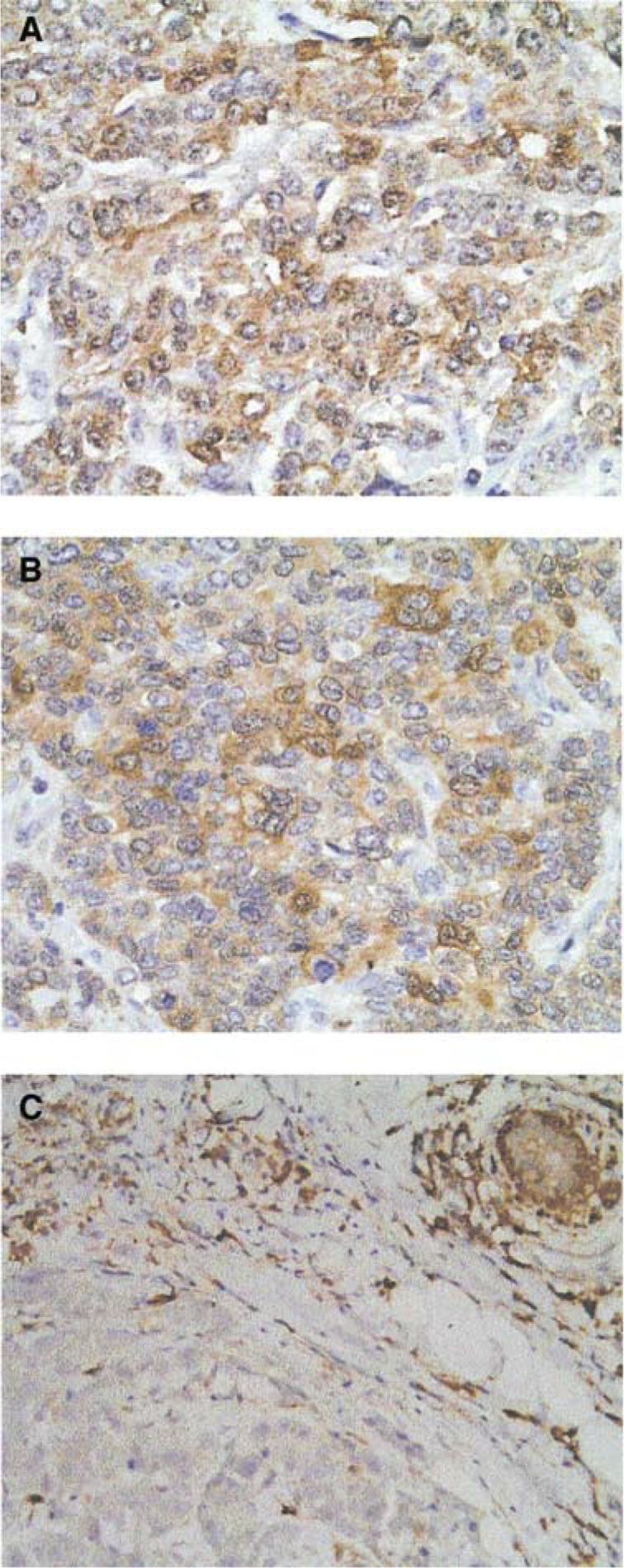
Protein kinase C alpha immunohistochemical staining of representative patient tumours. (**A**, **B**) Matched primary and recurrent biopsies from patient #10. **(C)** Primary biopsy from patient #4 exhibiting no disease recurrence. Magnification × 40.

To determine the possible association of PKC*α* overexpression with the acquisition of TAM resistance, PKC*α* expression was assessed by immunohistochemical staining in two patient populations treated with TAM: patients remaining disease-free following TAM treatment and patients exhibiting disease recurrence. Representative immunohistochemical staining of patient tumours demonstrates the cytoplasmic localisation of PKC*α* (Figure 1

). Seven percent (one out of 15) of patients remaining disease-free exhibited moderate PKC*α* staining and the remaining 14 biopsies exhibited negative or weak positive PKC*α* immunostaining (Table 1

**Table 1:** PKC*α* immunostaining: patients remaining disease-free

**Patient no.**	**Age (y)**	**LN^a^+/total**	**Grade**	**Primary biopsy^b^**
1	87	10/19	2	1+
2	41	20/21	3	1+
3	51	0/13	1	0
4	45	0/18	2	0
5	54	0/0	1	0
6	46	7/18	3	0
7	40	0/18	2	0
8	35	0/0	2	0
9	53	0/0	2	0
10	69	0/0	2	1+
11	58	0/0	3	0
12	48	8/20	2	0
13	50	20/21	3	0
14	72	0/0	1	2+
15	56	0/24	3	1+

PKC*α*=protein kinase C alpha.

a LN=lymph node;

b 0=negative; 1+=<10% staining; 2+=10–30% staining; 3+=>30% staining.

). Seventy-three percent (11 out of 15) of patients exhibiting disease recurrence showed moderate to intense PKC*α* immunoreactivity in both the primary and recurrent tumour (Table 2

**Table 2:** PKC*α* immunostaining: patients remaining disease-free

**Patient no.**	**Age at diagnosis (y)**	**LN^a^+/total**	**Grade**	**Primary biopsy^b^**	**Recurrent biopsy^b^**
1	45	0/28	3	0	0
2	36	17/17	3	0	1+
3	49	0/0	3	0	1+
4	36	0/13	3	0	1+
5	40	20/24	3	3+	2+
6	48	0/18	3	3+	2+
7	42	0/11	2	3+	3+
8	60	8/16	3	3+	3+
9	37	0/15	3	2+	2+
10	41	2/14	3	3+	3+
11	54	0/0	2	2+	2+
12	56	18/26	3	2+	2+
13	44	0/0	2	2+	2+
14	46	4/8	3	2+	2+
15	30	2/9	3	2+	1+

PKC*α*=protein kinase C alpha.

a LN=lymph node;

b 0=negative; 1+=<10% staining; 2+=10–30% staining; 3+=>30% staining.

). These results suggest that PKC*α* overexpression is more frequent in primary tumours of patients who experience disease recurrence compared with patients who remain disease-free. Furthermore, PKC*α* overexpression may be predictive of TAM treatment failure, since expression is high in the primary biopsy and does not increase in the second biopsy following TAM exposure. However, the sample size of this study is small, and the majority of patients who relapse are younger (mean age 44 years) and have tumours of higher grade compared with the disease-free patients (mean age 54 years).

## DISCUSSION

The implications for the use of PKC*α* overexpression as a predictive tool for improved therapeutic options are based on our T47D/PKC*α* xenograft model system demonstrating that E2 induces tumour regression (Chisamore *et al*, 2001). Other examples of E2-induced regression in both tumours and breast cancer cell lines have been described. Similar to the T47D/PKC*α* tumour, the T61 tumour derived from a primary breast cancer (Brunner *et al*, 1996) is ovarian-independent and E2 is inhibitory. However, in contrast to the T47D/PKC*α* tumour, growth of T61 is inhibited by TAM. A cyclical model of hormonal response in MCF-7 tumours has been described wherein after 1 year of TAM treatment, tumours are stimulated by both E2 and TAM, but following 5 years of treatment, E2 causes tumour regression (Yao *et al*, 2000). We have reported that PKC*α* is overexpressed in MCF-7 tumours after different periods of exposures to TAM (1 year and 5 years) (Chisamore *et al*, 2001). Both the MCF-7-derived E8CASS cell variant (Sonnenschein *et al*, 1994) and the MCF-7 long-term oestrogen deprived (LTED) cells (Masamura *et al*, 1995; Shim *et al*, 2000) were derived by long-term oestrogen deprivation and undergo apoptosis in response to E2 (Song *et al*, 2000). A recent report demonstrated that E2-induced apoptosis is likely triggered via a Fas-mediated mechanism (Song *et al*, 2001). T47D L(hE) cells described by Fernandez *et al* (1998) were cultured in E2-deficient media long term and also exhibit E2-induced growth inhibition. However, these cells were found to express elevated ER levels with a C→A transversion resulting in an H513N amino-acid change in the ligand-binding domain.

These findings, taken together with our previous observation that E2 has a novel antitumour effect on T47D/PKC*α* breast tumours (Chisamore *et al*, 2001), may have important therapeutic implications in the management of breast cancer patients. Elevated tumour expression of PKC*α* may predict TAM treatment failure and indicate that an oestrogenic compound may be more efficacious than TAM and perhaps an aromatase inhibitor. A recent updated analysis of diethylstilbestrol (DES) *vs* TAM for the treatment of postmenopausal metastatic breast cancer indicates that there is survival advantage for women on DES compared to women on TAM (Peethambaram *et al*, 1999); however, the basis of this survival advantage remains unknown. Perhaps, preselection of a subset of patients who overexpress PKC*α* may improve upon the outcome of treatment with DES. We are in the process of expanding these studies in a larger population of patients who are matched by age and stage to substantiate these preliminary findings. Examination of a larger patient series will also allow us to address the relations between ER status and PKC*α* expression as well as between the intensity of PKC*α* staining and time to disease recurrence.

## References

[bib1] Borner C, Wyss R, Regazzi R, Eppenberger U, Fabbro D (1987) Immunological quantitation of phospholipid/Ca^2+^-dependent protein kinase of human mammary carcinoma cells: inverse relationship to estrogen receptors. Int J Cancer 40: 344–348 3623717 10.1002/ijc.2910400310

[bib2] Brunner N, Spang-Thomsen M, Cullen K (1996) The T61 human breast cancer xenograft: an experimental model of estrogen therapy of breast cancer. Breast Cancer Res Treat 39: 87–92 8738608 10.1007/BF01806080

[bib3] Chisamore MJ, Ahmed Y, Bentrem DJ, Jordan VC, Tonetti DA (2001) Novel antitumor effect of estradiol in athymic mice injected with a T47d breast cancer cell line overexpressing protein kinase c alpha. Clin Cancer Res 7: 3156–3165 11595710

[bib4] Dumont JA, Bitonti AJ, Wallace CD, Baumann RJ, Cashman EA, Cross-Doersen DE (1996) Progression of MCF-7 breast cancer cells to antiestrogen-resistant phenotype is accompanied by elevated levels of AP-1 DNA-binding activity. Cell Growth Differ 7: 351–359 8838865

[bib5] Fernandez P, Wilson C, Hoivik D, Safe SH (1998) Altered phenotypic characteristics of T47D human breast cancer cells after prolonged growth in estrogen-deficient medium. Cell Biol Int 22: 623–633 10452832 10.1006/cbir.1998.0303

[bib6] Gordge PC, Hulme MJ, Clegg RA, Miller WR (1996) Elevation of protein kinase A and protein kinase C activities in malignant as compared with normal human breast tissue. Eur J Cancer 32A: 2120–2126 9014755 10.1016/s0959-8049(96)00255-9

[bib7] Johnston SR, Lu B, Scott GK, Kushner PJ, Smith IE, Dowsett M, Benz CC (1999) Increased activator protein-1 DNA binding and c-Jun NH2-terminal kinase activity in human breast tumors with acquired tamoxifen resistance. Clin Cancer Res 5: 251–256 10037172

[bib8] Masamura S, Santner SJ, Heitjan DF, Santen RJ (1995) Estrogen deprivation causes estradiol hypersensitivity in human breast cancer cells. J Clin Endocrinol Metab 80: 2918–2925 7559875 10.1210/jcem.80.10.7559875

[bib9] O'Brian C, Vogel VG, Singletary SE, Ward NE (1989) Elevated protein kinase C expression in human breast tumor biopsies relative to normal breast tissue. Cancer Res 49: 3215–3217 2720675

[bib10] Peethambaram PP, Ingle JN, Suman VJ, Hartmann LC, Loprinzi CL (1999) Randomized trial of diethylstilbestrol *vs* tamoxifen in postmenopausal women with metastatic breast cancer. An updated analysis. Breast Cancer Res Treat 54: 117–122 10424402 10.1023/a:1006185805079

[bib11] Schiff R, Reddy P, Ahotupa M, Coronado-Heinsohn E, Grim M, Hilsenbeck SG, Lawrence R, Deneke S, Herrera R, Chamness GC, Fuqua SA, Brown PH, Osborne CK (2000) Oxidative stress and AP-1 activity in tamoxifen-resistant breast tumors *in vivo*. J Natl Cancer Inst 92: 1926–1934 11106684 10.1093/jnci/92.23.1926

[bib12] Shim WS, Conaway M, Masamura S, Yue W, Wang JP, Kmar R, Santen RJ (2000) Estradiol hypersensitivity and mitogen-activated protein kinase expression in long-term estrogen deprived human breast cancer cells *in vivo*. Endocrinology 141: 396–405 10614662 10.1210/endo.141.1.7270

[bib13] Song R, McPherson R, Yue W, Wang J, Santen R (2000) Estrogen induces apoptosis in human breast cancer cells adapted to long term estrogen deprivation. In American Association for Cancer Research Vol. 41, pp 427 San Francisco, CA

[bib14] Song RX, Mor G, Naftolin F, McPherson RA, Song J, Zhang Z, Yue W, Wang J, Santen RJ (2001) Effect of long-term estrogen deprivation on apoptotic responses of breast cancer cells to 17beta-estradiol. J Natl Cancer Inst 93: 1714–1723 11717332 10.1093/jnci/93.22.1714

[bib15] Sonnenschein C, Szelei J, Nye TL, Soto AM (1994) Control of cell proliferation of human breast MCF7 cells; serum and estrogen resistant variants. Oncol Res 6: 373–381 7894086

[bib16] Tonetti DA, Chisamore MJ, Grdina W, Schurz H, Jordan VC (2000) Stable transfection of protein kinase C alpha cDNA in hormone-dependent breast cancer cell lines. Br J Cancer 83: 782–791. doi:10:1054/bjoc.2000.1326 10952784 10.1054/bjoc.2000.1326 PMC2363523

[bib17] Yao K, Lee ES, Bentrem DJ, England G, Schafer JI, O'Regan RM, Jordan VC (2000) Antitumor action of physiological estradiol on tamoxifen-stimulated breast tumors grown in athymic mice. Clin Cancer Res 6: 2028–2036 10815929

